# Attack-Aware IoT Network Traffic Routing Leveraging Ensemble Learning

**DOI:** 10.3390/s22010241

**Published:** 2021-12-29

**Authors:** Qasem Abu Al-Haija, Ahmad Al-Badawi

**Affiliations:** 1 Department of Computer Science/Cybersecurity, Princess Sumaya University for Technology, Amman 11941, Jordan; 2 Department of Homeland Security, Rabdan Academy (RA), Abu Dhabi 22401, United Arab Emirates; aalbadawi@ra.ac.ae

**Keywords:** cybersecurity, Internet of Things, network layer, intrusion detection, intrusion classification, ensemble learning

## Abstract

Network Intrusion Detection Systems (NIDSs) are indispensable defensive tools against various cyberattacks. Lightweight, multipurpose, and anomaly-based detection NIDSs employ several methods to build profiles for normal and malicious behaviors. In this paper, we design, implement, and evaluate the performance of machine-learning-based NIDS in IoT networks. Specifically, we study six supervised learning methods that belong to three different classes: (1) ensemble methods, (2) neural network methods, and (3) kernel methods. To evaluate the developed NIDSs, we use the distilled-Kitsune-2018 and NSL-KDD datasets, both consisting of a contemporary real-world IoT network traffic subjected to different network attacks. Standard performance evaluation metrics from the machine-learning literature are used to evaluate the identification accuracy, error rates, and inference speed. Our empirical analysis indicates that ensemble methods provide better accuracy and lower error rates compared with neural network and kernel methods. On the other hand, neural network methods provide the highest inference speed which proves their suitability for high-bandwidth networks. We also provide a comparison with state-of-the-art solutions and show that our best results are better than any prior art by 1~20%.

## 1. Introduction

It can be argued that the world entered the digital age in the 1970s due to the advent of personal computers and computer networks, both facilitating the ability to create, store, and transfer information freely and swiftly. Since then, more computing devices and interconnection technologies have emerged to facilitate large computer networks such as the Internet. And over the last decade, driven by advancements in cheap electronic sensory devices, larger computing capacity per area, ultrafast computer networks, and practical artificial intelligence, the world is entering into another era where both people and things are getting interconnected, what is usually referred to nowadays as the Internet of Things (IoTs) [1,2].

**Figure 1:**
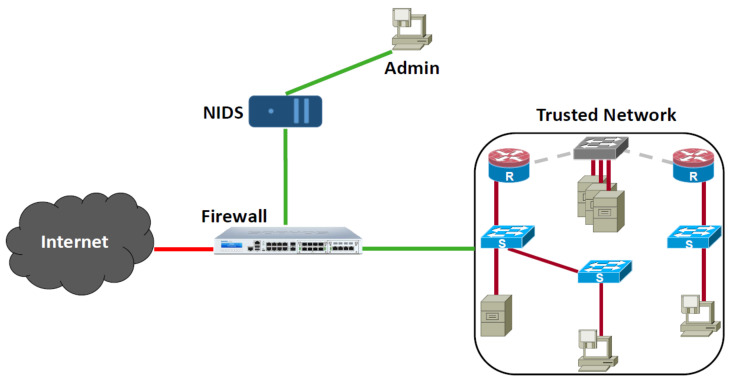
NIDS typical deployment in computer networks.

Due to the significant benefits such technologies bring into its adopters, they have been deployed in several domain areas such as healthcare [3], finance and banking [4], national security [5], military [6], disease control [7], and many more [8,9]. Some of these deployments are highly critical when service disruption might be intolerable. As such, defending these networks against malicious attacks cannot be overstated. A very important and indispensable defense tool is the Network Intrusion Detection System (NIDS), which monitors network traffic for anomalous behaviors [10,11] (See Figure 1: NIDS typical deployment in computer networks.). Upon the detection of intrusion or anomalous activity, NIDS reports the incident to the network administrator for immediate action or further investigation. NIDSs are usually deployed in two operation modes: (1) online wherein the NIDS is deployed at the network gateway and monitors the flowing traffic as it enters the network and (2) offline wherein the NIDS analyzes archived network traffic [12].

The most widely used and performant NIDSs are those that employ pattern recognition capabilities [13,14,15]. In these NIDSs, the communication network is monitored to build profiles for normal and abnormal activities. Several methods can be used to construct these profiles such as statistical analysis (also known as discriminant analysis) [16], correlations [17], and similarity measures [18]. Modern techniques for pattern recognition include machine and deep learning [19], which proved to provide high performant accuracy, lower false positive and negative rates, and relative ease of usage. In this work, we analyze the performance of several machine-learning techniques for constructing NIDSs. Specifically, we use six different algorithms: Ensemble Boosted Trees (EBT) [20], Ensemble RUSBoosted Trees (ERT) [21], Ensemble Subspace KNN (ESK), Shallow Neural Network (SNN), Bilayered Neural Network (BNN), and Logistic Regression Kernel (LRK) to construct an NIDS. To the best of our knowledge, this is the first work that employs the aforementioned ensemble learning methods in IoT NIDS. We model the intrusion detection problem as a multilabel, supervised-learning problem in which a set of data points and associated labels are given. Each machine-learning method is used to build a classification function that assigns a label for each input data point.

The choice of the aforementioned machine-learning techniques was driven by the maturity and ubiquity of the techniques, high accuracy rates, and efficient inference speed. Our choice was also driven by a less significant factor, that is, the consideration of more diversified methods in terms of operation mode and underlying assumptions about the dataset to provide more insights about this particular NIDS problem. Of particular interest are ensemble learning methods which proved to be more performant than single-model techniques in several machine-learning problems [22,23,24,25,26,27]. Conforming with the literature, our experimental analysis shows the superiority of these methods over other single-model techniques. It should be remarked that the aforementioned criteria for algorithm selection are not standard, but rather, are our recommendations based on experience. It is well known that the choice of machine-learning algorithms for a given problem statement is governed by the no-free-lunch law; therefore, a trial-and-error approach is usually the safest approach to follow in algorithm selection. We believe that a diversified space of algorithms in terms of assumptions (parametric vs. non-parametric), explainability (white box vs. black box), execution overhead, and data engineering efforts are key factors that should be considered for algorithm selection. In Table 1 below, we provide the main factors affecting our choice of the aforementioned algorithms.

We use two datasets, the celebrated NSL-KDD [28] and a distilled version [22] of the Kitsune dataset [12] (hereafter referred to as distilled-Kitsune-2018). In distilled-Kitsune-2018, two network deployments were studied in [12]: (1) a surveillance network with four IP cameras (SNC-EM602RC, SNC-EM600, SNC-EB600, and SNC-EB602R) was put under eight attacks that affected data confidentiality, data integrity, and the availability of the network, and (2) an IoT wi-fi network that included three personal computers and nine IoT devices (a thermostat, baby monitor, webcam, two doorbells, and four security cameras with one of the latter infected by the Mirai botnet malware [23]). Collectively, the distilled-Kitsune-2018 dataset is, therefore, a 10-class labeled dataset (nine attacks and one normal) with 115,391 data points. Each data point includes 115 numerical features (excluding the label). The features include 23 different statistics of the bandwidth of inbound/outbound traffic, packet rates, interpacket delays, channel and TCP/UDP socket information captured over 5-time windows (hence 115 features overall). We remark that the distilled-Kitsune-2018 dataset bundles the aforementioned attacks independently. In our analysis, we combine all the attacks in one dataset that is used as input for each of the six supervised machine-learning methods used in our study.

Our evaluation methodology employs standard machine-learning evaluation metrics, such as classification accuracy, precision, recall, error rates (type-1 and type-2 errors) and inference speed. To preview, it was found that ensemble methods (EBT, ESK, and ERT) provide the highest accuracy rates (more than 98%) and, accordingly, the lowest error rates. In terms of inference speed, the neural network methods (SNN and BNN) provide the lowest inference overhead. On the other hand, the kernel method (LRK) showed the worst performance, both in terms of accuracy and inference speed.

This work provides a detailed comparative analysis of various intrusion detection schemes for detecting and classifying several IoT network intrusions. Our main contributions can be summarized as follows:

1. We design and implement a multipurpose, lightweight, and highly accurate anomaly-based IoT NIDS using various machine-learning methods.
2. We characterize and evaluate the performance of three ensemble learning techniques (EBT, ESK, and ERT) for IoT NIDSs using the NSL-KDD and distilled-Kitsune-2018 datasets.
3. We provide a thorough empirical analysis of six different supervised machine-learning methods using eight standard performance evaluation metrics.
4. We also compare our results with state-of-the-art NIDS solutions and show that our ensemble-based NIDS is better than any prior art by 1–20%.

The rest of the paper is organized as follows. In Section 2, we briefly review the state-of-the-art of machine-learning-based NIDSs. The system model is presented in Section 3. Evaluation methodology, experiments, and results are provided in Section 4. Finally, Section 5 concludes the work by providing the key takeaways of our study and lays out future works.

## 2. Related Work

The employment of machine learning in developing NIDSs dates to the 1990s [29,30,31]. In this section, we limit our review to contemporary studies in the past five years. Some older studies are included for their importance and monumental results.

In [32], the authors surveyed statistical, machine learning, and data mining methods for constructing NIDSs in Software Defined Networks (SDNs). Based on their study, the authors recommend the suitability of machine-learning methods over other methods due to their flexibility, lightweight inference overhead, and high accuracy rates. In [33], the authors presented a two-stage deep and machine-learning approach to tackle the network intrusion detection problem. In the first phase, they use a dimensionality reduction framework to strip down more than 88% of the input feature space without affecting the accuracy rate significantly. The main reason for doing that is to reduce the training time and inference overhead. They reported that Random Forests (RF) achieved 0.996 F-measure (which is a performance criterion that relates precision and recall) on the CICIDS2017 dataset. A similar approach was also followed in [34]. The authors found that ANN with wrapper feature selection outperformed Support Vector Machines (SVMs) on the NSL-KDD dataset. As will be shown later, our analysis conforms with this result for the distilled-Kitsune-2018 dataset.

In a recent work, the authors in [35] tried to provide a standard feature set for NIDSs. The authors collected two NetFlow-based feature sets and converted four commonly used NIDS datasets (See Table 2) to conform with the collected datasets. Their main goal was a trial towards standardizing NIDS evaluation datasets. The Extra Trees ensemble learning was used to evaluate the performance of the compiled datasets and proposed feature set. Their best result for the multiclass classification problem was 98% of classification accuracy. Later we show that our best result outperforms their results using EBT, ESK, and ERT ensemble classifiers.

A recent and relevant study to our work was proposed in [36]. The authors presented a statistical-analysis-based NIDS that employs Beta Mixture Model (BMM) and a Corrs entropy model. Their proposed solution was evaluated on the same dataset used in this research, that is, the Kitsune dataset among the other two datasets. The best accuracy result reported in their study was 99.2%, lower than our best result by 0.60%.

In [37], the authors proposed a more sophisticated approach that combined blockchain technology and machine learning. The blockchain technology is used to add a privacy-preserving capability to the NIDS. They also used PCA for dimensionality reduction of data points extracted from the ToN-IoT and BoT-IoT. Their model recorded 97.7% of classification accuracy. In [38], the authors provided another ensemble-learning, voting classifier-based NIDS that adopted the Decision Trees (DT), Naïve Bayes (NB), Random Forest (RF), and k-Nearest Neighbors (k-NN). They test their NIDS on the Ton-IoT datasets, achieving accuracy results with high variance, ranging from 58% to 100% on each dataset individually. Their system did not seem to perform well when they evaluated it on the combined dataset and reported accuracy results ranging from 75% to 76% in the multiclass setting.

In [39], the authors developed an IoT cyberattack detection and classification system, making use of a shallow convolutional neural network. They evaluated their model on the NSL-KDD dataset, recording maximum accuracy of 99.3% and 98.2% for the two-class (normal vs. attack) and multi-class (normal, Probe, R2L, U2R, and DDos) classifiers, respectively.

The author in [40] used tree-based and ensemble methods to build a NIDS using the NSL-KDD dataset, achieving classification accuracy of 97% using the XGBoost algorithm. They also evaluated the performance of some unsupervised learning algorithms, such as the Expectation-Maximization, reporting less than 67% classification accuracy, which showed superiority among the supervised methods in comparison to the unsupervised methods in their particular settings. In [41], the authors introduced the Ton-IoT dataset, a contemporary dataset for developing and testing IDSs. Several machine-learning methods were used such as LR, LDA, RT, RF, and NB. Their best classification accuracy result was reported on the weather dataset using CART and achieving 87%.

The authors in [42] presented a distributed modular solution (EDIMA) that employs machine-learning algorithms for edge devices’ traffic classification. EDIMA can be used towards the detection of IoT malware network activity in large-scale networks. They evaluated the performance of their model using several evaluation metrics including classification accuracy, classification precision, and classification sensitivity, recording 94.44%, 92.00%, and 100.0%, respectively.

We provide a comparative summary of state-of-the-art NIDS solutions for IoT communication networks employing diverse machine-learning techniques in Table 2. The table recaps the reference number, the machine-learning methods, the employed datasets, and the target attacks (classes) for every state-of-the-art NIDS.

## 3. System Modeling

Cyberattacks are meant to deliberate an exploitation of system resources to compromise necessary data and utilize the system in illegal usage [46]. Cyberattacks usually are launched against the victim system to violate the major security services, such as confidentiality, integrity, and availability. Currently, cyberattacks are available in different forms such as ransomware, malware, adware, reconnaissance, denial of service, and others [47,48]. In this paper, we develop a cyberattacks detection system making use of efficient machine-learning techniques to provide an accurate, precise, and sensitive prediction process. To develop and evaluate the proposed system, a high-performance computing platform (hardware and software) has been used and it is described in Table 3, which states the simulation environment for system development and validation.

**Figure 2:**
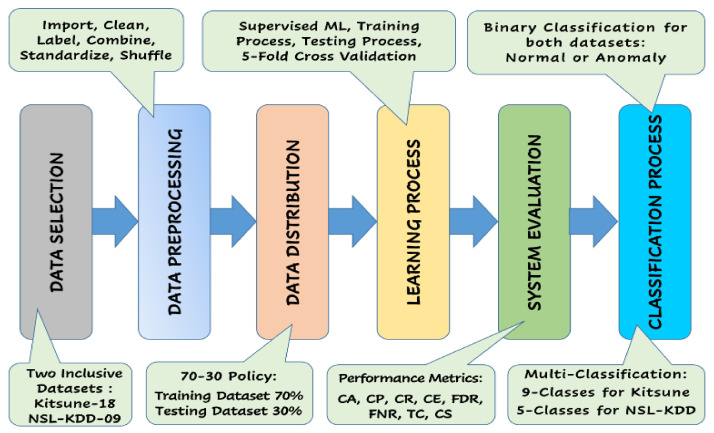
Workflow Diagram for attack-aware IoT network traffic routing via ML techniques.

We have decomposed the proposed system to be developed into seven consecutive subsystems (modules). Figure 2 illustrates the workflow diagram for the proposed attack aware IoT network traffic routing via ML techniques. The diagram provides the computational process, starting from obtaining the IoT network traffic routing packets, passing through the traffic data preparation, distribution, and learning, before it provides the proper predictive output for every data packet.

**Figure 3:**
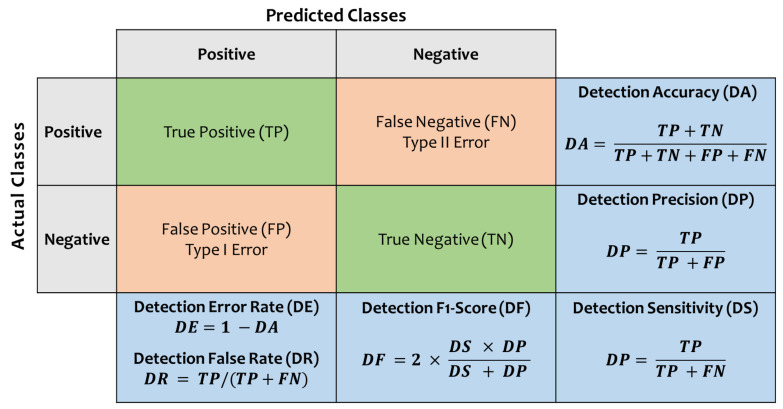
Confusion matrix with other performance evaluation measures.

Specifically, the proposed system is composed of the following consecutive subsystems/modules:

- **Data Selection Subsystem:** This subsystem involves obtaining a representative dataset that can be applied to the proposed NIDS to express the IoT network traffic routing. To build a comparative study and gain more insight into the solution approach, Distilled-Kitsune-2018 dataset [12] and NSL-KDD dataset [28] have been employed at this stage. These two datasets were selected since they are both comprehensive, publicly available, well-established as they are used in several reputable research studies, have a fairly large number of samples (~150,000 network traffic records for each of them), and they cover wide spectrum attack vectors for IoT in specific and general computer networks. The distilled-Kitsune-2018 dataset, which was collected by Mirsky et al. (2018), recorded a total of ~150,000 network traffic records of normal traffic and nine different attacks targeting the violation of the three main security services known as CIA triad (confidentiality, integrity, availability) including: two reconnaissance attacks (OS Scan attack and Fuzzing attack), three man-in-the-middle (MitM) attacks (video injection attack, ARP attack, and active wiretap attack), three denial-of-service (DoS) attacks (SSDP Flood attack, SYN DoS attack, and SSL renegotiation attack), and one botnet malware attack (Mirai attack) [12]. On the other hand, NSL-KDD [28], which is a newer and reduced version of the original KDD’99 dataset, was developed by the Defense Advanced Research Projects Agency (DARPA) and has been revised to include more up-to-date and nonredundant attack records with different levels of difficulty. The NSL-KDD dataset is available as a two-class traffic dataset (normal vs. anomaly) and as a multiclass traffic dataset that includes attack-type labels and a difficulty level (normal, DoS attacks, Probe attacks, Root-to-Local (R2L) attacks, and User-to-Root (U2R) attacks). In both cases, it comprises a total of ~150,000 samples, each with 43 attributes, such as duration, protocol, and service. The summary of the distilled-Kitsune-2018 and NSL-KDD dataset distribution is provided in Table 4 below.
- **Data Preprocessing Subsystem:** This subsystem involves the preparation of the dataset to be fed through the machine-learning processes, which is initiated when the data is imported from the CSV files though MATLAB containers to be stored and processed via MATLAB tables. The data then passes through a cleaning process by excluding the untrainable features, filling the unimportable data cells with zero values, filling the empty data cells with zero values, and unifying the number of extracted features for all traffic datasets. Then, all datasets are labeled using categorical labeling and then encoded into integer encoding (0–9). Thereafter, the data records from all attacks’ datasets are combined to form one large and comprehensive dataset containing all types of traffic (normal, OS Scan, Fuzzing, Video Inj, ARP, Wiretap, SSDP F, SYN DoS, SSL R, and Mirai). After that, all numerical data values are standardized to enhance the classifier task, and finally, all records are randomly shuffled to eliminate any biasing in the classifier process.
- **Data Distribution Subsystem:** This subsystem involves the random division (using the DivideRand Algorithm [49]) of the preprocessed dataset into the training dataset and the validation (testing) dataset. We have used the policy of 70%:30% distribution for training dataset:testing dataset, respectively. Also, the data has been randomly distributed into five different 70%:30% distributions to accommodate the 5-fold cross validation process [49] performed at the learning stage.
- **Learning Process Subsystem:** This subsystem involves the development and employment of the different machine-learning models to train and test/validate the considered datasets. Supervised machine-learning methods are usually employed to develop solutions for regression [50], prediction [51], and classification [52]. We have employed six different supervised ML schemes including Ensemble Boosted Trees (EBT) [53], Ensemble Subspace kNN (ESK) [54], Ensemble RUSBoosted Trees (ERT) [55], Shallow Neural Network (SNN) [56], Bilayered Neural Network (BNN) [57], and Logistic Regression Kernel (LRK) [58]. The exploited ML models are summarized in Table 5 below.
- **System Evaluation Subsystem**: This subsystem involves the evaluation of the performance of the proposed NIDS using several quality indication factors [59], including validation accuracy (CA), validation precision (PR), validation recall (RC), misclassification rate (MCR), false discovery rate (FDR), false negative rate (FNR), total validation cost (TC) in terms of the number of misclassified samples, and classification speed (CS) in terms of the number of observations per second. Such quality indication factors are also used to opt the most advantageous model to employ for attack-aware IoT network traffic routing detection/classification. Moreover, they are also used to contrast the results of our best NIDS with other state-of-the-art models in the same area of study. A summary of the evaluation metrics are depicted in Figure 3 below.
- **Classification Process Subsystem:** This subsystem involves the categorization of the traffic records into a binary-classification process to either normal vs. anomaly (attack) or to a multiclassification process to a dedicated traffic category in the Kitsune dataset {normal, OS Scan attack, Fuzzing attack, Video Inj attack, ARP attack, Wiretap attack, SSDP F attack, SYN DoS attack, SSL R attack, and Mirai attack}, and in the NSL-KDD dataset {normal, DoS attacks, probe attacks, R2L attacks, and U2R attacks}.

## 4. Results and Discussion

In this section, we provide the experimental results obtained during the simulation and system evaluation stage. Table 6 provides a summary of the system evaluation results of the six ML based models (EBT, ESK, ERT, BNN, SNN, and LRK) in terms of four quality indication-factors (CA, MCR, TC, and CS) for the multiclass classification. According to the table, it can be clearly observed that the EBT model is the optimal predictive model to be used as an attack-aware for IoT network traffic routing, scoring 99.8% of classification accuracy with the least number of misclassified records (TC) and with a very satisfiable prediction speed (requiring 11.11 µsec/single-prediction). Another noticeable predictive model is the BNN model which is featured as the fastest predictive model scoring 290,000 observations/sec (requiring 3.44 µsec/single prediction) and with a very satisfiable prediction accuracy of 97.5%. Moreover, though ESK recorded a superior prediction accuracy (99.4%), it has a high prediction overhead requiring a long time to provide prediction for traffic records, recording 41 observations/sec (requires 24.4 milliseconds for each single prediction). These results exhibit the improved overall prediction performance evaluation for the models employing the Kitsune dataset over the models employing NSL-KDD. This seems to be rational as the Kitsune datasets have better dataset sample balancing and have much more features (116 features ≈ three times greater than the number of features in NSL-KDD), which provide better distinction of traffic records and improve classification accuracy and precision.

**Figure 4:**
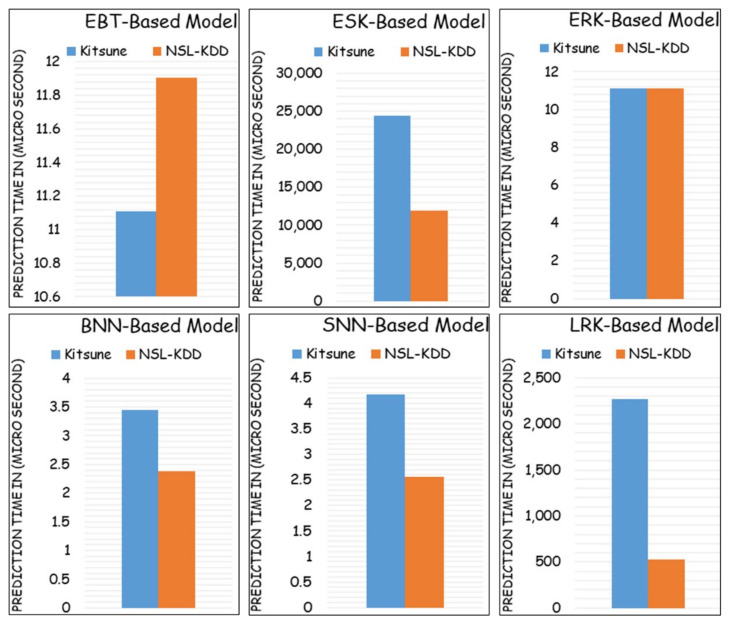
Timing complexity of both datasets using the six above mentioned ML models.

In addition, Figure 4 compares the prediction time complexity computed in micro-seconds of both the Kitsune and NSL-KDD datasets using the six employed machine-learning schemes. As can be clearly seen, the prediction time for NSL-KDD seems to be faster than the prediction time for the Kitsune dataset in four models (i.e., ESK-based model, BNN-based model, SNN-based model, and LRK-based model) and slower in one predictive model (i.e., EBT-based model) and has equal prediction time in one model (i.e., ERK-based model) and slower in one predictive model (i.e., EBT-based model). Overall, the BNN-based predictive model seems to be the fastest predictive model with only 2.5–3.5 µseconds, whereas the ESK-based predictive model seems to be the slowest predictive model with 12–24 milliseconds. However, to obtain the optimum predictive model considering all the aforementioned performance metrics, one will absolutely select the EBT-based model, which provides the traffic prediction with only 11–11.8 µseconds, scoring the maximum prediction accuracy and the minimum misclassification rate.

**Figure 5:**
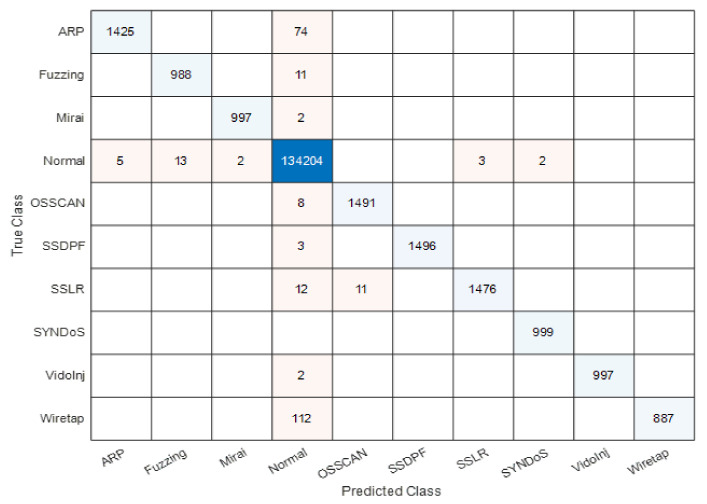
Confusion matrix for Ensemble Boosted Trees (EBT) classifier.

Based on the results reported in Table 6 and the timing complexity figure (Figure 4) as well as their corresponding discussion/analysis, EBT has been selected to detect and classify the IoT network traffic records of distilled-Kitsune-2018 dataset. Therefore, the rest of the discussion of this paper will focus on the EBT-based model for the distilled-Kitsune-2018 dataset. Indeed, EBT was able to perform the detection stage of the two-class classifier (normal vs. attack) with 100% accuracy. EBT has also been exploited to provide detection (normal vs. attack) for every individual attack dataset with 99.9–100% accuracy. Therefore, we have focused our reported results in the tables and figures for the multiclass classification. Thus, EBT is elected with confident. Therefore, the next results, discussions, and comparisons will focus on the EBT-based model. For instance, Figure 5 depicts the multiclass confusion matrix results for the EBT model. Accordingly, most of the traffic records were truly predicted as true positives (TP) and true negatives (TN) with minor numbers recorded for Type I errors (Fales positives-FP) and Type II errors (False negatives-FN).

**Figure 6:**
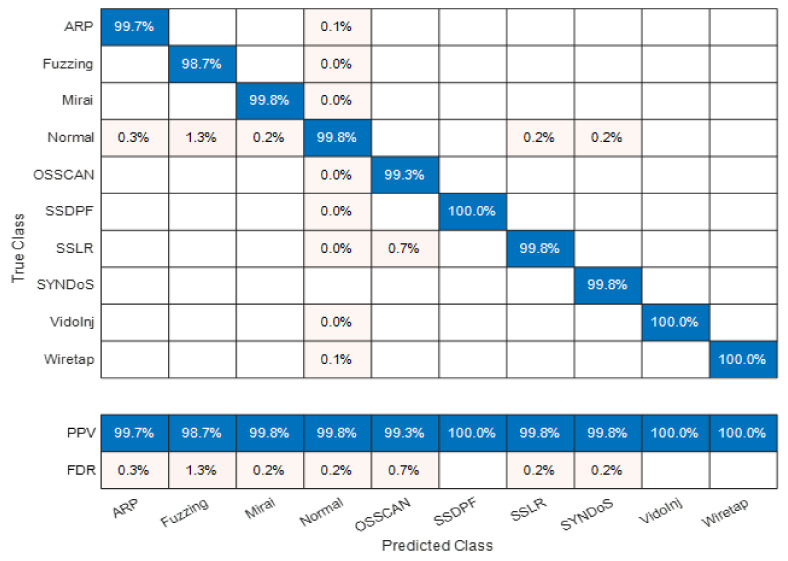
Matrix of PPV vs. FDR for each individual class using EBT classifier.

Moreover, Figure 6 investigates the matrix of the positive predictive values (PPV)—also known a predictive precision—and false discovery rates (FDR)—also known as predictive imprecision—for each individual class using the EBT classifier. As observed from the figure, all attack classes are precisely predicted with the maximum PPV proportion of 100% recorded for the SSDPF attack-type class and the least PPV proportion of 98.7% recorded for the fuzzing reconnaissance attack-type class. Overall, the average precision (PPV or PR) involving all classes of the dataset records is 99.69%, which shows that in addition to being very accurate, the EBT ensemble model is also considered very precise in providing both attack detection and classification for the IoT network traffic routing packets.

**Figure 7:**
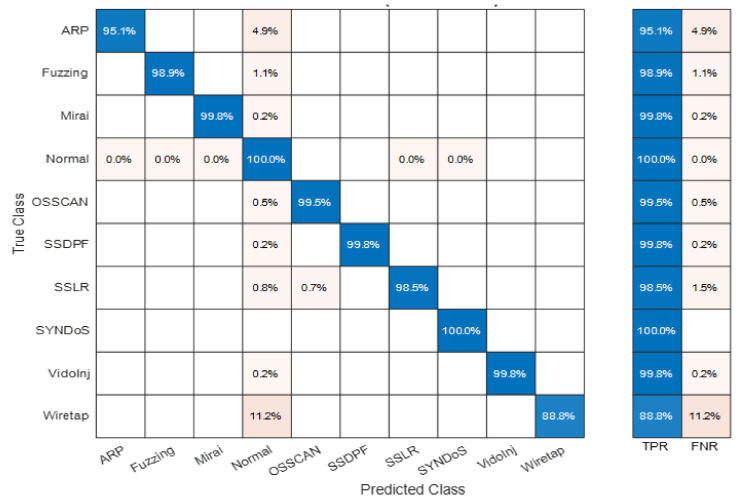
Matrix of TPR vs. FNR for each individual class using EBT classifier.

Additionally, Figure 7 investigates the matrix of true negative values (TPR)—also known as predictive recall/sensitivity—and falls negative rates (FNR)—also known as predictive insensitivity—for each individual class using the EBT classifier. As observed from the figure, all attack classes are sensitively predicted with maximum TPR proportion of 100% recorded for the SYN-DoS attack-type class and the least TPR proportion of 88.8% recorded for the active wiretap MitM attack-type class. Overall, the average sensitivity (TPR or RC) involving all classes of the dataset records is 98.1%, which shows that in addition of being very accurate and precise, the ETB ensemble model is also considered very sensitive in providing both attack detection and classification for the IoT network traffic routing packets.

Table 7 provide a summary of quality indication factors for the EBT-based model in terms of accuracy (CA), precision (PR), recall (RC) misclassification rate (MCR), false discovery rate (FDR), false negative rate (FNR), and classification speed (CS).

Lastly, Table 8 contrasts the performance outcomes obtained for the best of our ensemble-based attack-aware IoT network traffic routing systems (specifically, the EBT-based model) with the existing ML-based IoT attack-aware detection systems stated in the literature. The comparison considers five factors including the utilized supervised machine-learning technique (ML Model), the number of output classes of the classification or detection system (No. of Classes), the proportion of classification or detection accuracy (ACC%), the proportion of positive predictive value (PPV%), and the proportion of true positive rate (TPR%). Also, nine intelligent IoT-IDS-systems are deemed in this assessment as engaging diverse supervised ML systems containing: Extremely Randomized Trees (XRT) Classifier [35], Statistical Learning (STL) Classifier [36], eXtreme Gradient Boosting (XGB) Classifier [37,40], Hybrid ML Scheme combining decision trees, random forests, and Naïve bays algorithms (HYB) Classifier [38], shallow convolutional neural networks (S-CNN) Classifier [39,60], Classification And Regression Trees (CART) Classifier [41], k-nearest neighbor (kNN) Classifier, and our best system employing ensemble boosted trees (EBT) Classifier. According to the information provided in the table, it can be clearly inferred that our model is prominent as it recorded the best performance results among all other schemes. However, some other systems seem to be proficient, such as the Ashraf et al. model [36], Kumar et al. [42], and Al-Haija et al. [39], employing STL classifier, XGB Classifier, and S-CNN classifier, respectively, as well as registering classification accuracy proportions of 99.20%, 97.81%, and 98.20% for three classes, ten classes, and five classes, respectively.

## 5. Conclusions

This paper provided the design, implementation, and evaluation of an anomaly-based IoT NIDS using machine-learning techniques. The intrusion detection problem was modeled as a supervised multiclass learning problem in which a classification function is learnt to map a set of labeled data points to 10 different classes. We used six different machine-learning methods that belong to ensemble learning, neural networks, and kernel methods to develop the NIDS models. Two real-world IoT datasets (distilled-Kitsune-2018 and NSL-KDD) were used to evaluate the performance of the proposed NIDSs using standard classification performance criteria. Our analysis showed that ensemble methods provide higher accuracy percentages and lower error rates. On the other hand, neural network methods showed satisfactory accuracy with superior inference speed, making them very suitable for lightweight NIDSs. We also compared the performance of our best results with state-of-the-art solutions and demonstrated higher accuracy rates. We believe that this work provides different flavors of IoT NIDSs that suit different application requirements. In highly sensitive networks, we suggest using ensemble methods which exhibit high-accuracy profiles. On the other hand, neural networks might be more suitable for deployments in power-constrained environments. For future work, we believe that real-world deployment of the proposed NIDSs in different IoT networks (such as Internet of Drones and Vehicular Ad-hoc Network) is crucial for more accurate performance characterization and feasibility studies.

## Figures and Tables

**Table 1:** Machine-learning algorithms selection criteria.

Method	Assumptions	Explainability	Execution Time		Data Engineering
			Train	Test	
EBT, ERT	No assumptions about predictors or response variable	Intuitively explainable as rule-based knowledge system	Slow	Fast	Minimal effort
ESK	No assumptions	Intuitively explainable via similarity measures	Slow	Very slow	Minimal effort
SNN, BNN	No assumptions	Black box	Depends on network architecture	Fast	Medium effort
LRK	Linearity between predictors and response variable	Relatively explainable	Fast	Slow	Essential

**Table 2:** Comparison among some state-of-the-art NIDS solutions. ANN stands for Artificial Neural Networks, NB for Naïve Bayes, RF for Random Forests, SOM for Self-Organizing Maps, LDA for Linear Discriminant Analysis, QDA for Quadratic Discriminant Analysis, PCA for Principal Component Analysis, RT for Regression Trees, LR for Logistic Regression, U2R for User to Root, R2L for Remote to Local.

Ref.	Methods	Datasets	Attacks
[29]	ANN, SVM, NB, RF, SOM	NSL-KDD, KDD Cup 1999, CIC DOS, ADFA-LD12, UNSWNB15, WSN-DS	DDoS, flooding, U2R, Jamming
[33]	Auto-Encoder, RF, NB, LDA, QDA	CICIDS2017	DDoS, Heartbleed, SQL Injection, Botnet.
[34]	ANN, SVM	NSL-KDD	DDoS, R2L, U2R
[35]	Ensemble Learning (Extra Trees)	UNSW-NB15, BoT-IoT, ToN-IoT, CSE-CIC-IDS2018	DDoS, Botnet, Infiltration.
[36]	Statistical Analysis	Kitsune, ISCX, IoT network intrusion	Botnet, DDoS, MITM
[37]	XGBoost, PCA	ToN-IoT and BoT-IoT.	DDoS, Botnet, ransomware
[38]	Ensemble-based voting classifier	Ton-IoT	DDoS, Botnet, ransomware
[39]	Shallow CNN	NSL-KDD	Normal, DoS, Probe, R2L, U2R
[40]	XGBoost	NSL-KDD	SynFlood, UDP Flood, Smurf, and others
[41]	LR, LDA, RT, RF, and NB	TON-IoT	DDoS, Password, Backdoor, Ransomware
[42]	RF, KNN, NB	Simulated dataset	Satori, Reaper, Amnesia, Masuta, Mirai, others
[43]	Fuzzy C-means clustering and fuzzy interpolation	Kitsune	Botnet, MitM, DoS
[44]	Generative adversarial networks (GAN)	Kitsune, CICIDS	Artificially generated attacks
[45]	Extreme Value Analysis	Kitsune	Botnet, MitM, DoS

**Table 3:** Simulation Environment Specifications (Hardware and Software).

Item	Descriptions
Operation System	Windows 11, Edition 21H2, 64-bit operating system, x64-based processor
Processing Component	11th Gen Intel(R) Core(TM) i7-11800H @ 2.30 GHz⋯30 GHz
Computing Component	NVIDIA GeForce RTX 3050 Ti Laptop GPU@ 4 GBye
Memory Component	16.0 GB, DDR4 1.2v @ Memory Speed: 2933 MHz (PC4-23400)
Storage Component	500 GB Kingston NV1 M.2 (2280) PCIe NVMe Gen 3.0 (×4) SSD
Development Platform	MATLAB 2021b + Parallel Computing + Machine Learning Packages.

**Table 4:** Summary of Dataset Distribution: distilled-Kitsune-2018 and NSL-KDD.

Samples Distribution for Distilled-Kitsune-2018				
Attack		No. Training Packets	No. Normal Test Packets	No. Malicious Test Packets
OS Scan		6000	13,500	1499
Fuzzing		1200	9000	999
Video Inj.		4000	9000	999
ARP		6000	13,500	1499
Wiretap		4000	9000	999
SSDP F.		6000	13,500	1499
SYN DoS		1200	9000	999
SSL R.		6000	13,500	1499
Mirai		6000	9000	999
**Samples Distribution for NSL-KDD dataset**				
	**Normal**	**DoS**	**Probe**	**R2L**
Training	67,343	45,927	11,656	995
Testing	9711	7458	2754	2421
Total	77,054	53,385	14,410	3416

**Table 5:** Summary of System Development Parameters.

ML Model	Models Parameters
Ensemble Boosted Trees (EBT)	Ensemble method: AdaBoost, Learner type: Decision tree, Maximum number of splits: 20, Number of learners: 30, Learning rate: 0.1, 5-Fold Cross Validation.
Ensemble Subspace kNN (ESK)	Ensemble method: Subspace, Learner type: Nearest Neighbors, number of learners: 30, Subspace Dimension: 58, 5-Fold Cross Validation
Ensemble RUS_Boosted Trees (ERT)	Ensemble method: RUSBoost, Learner type: Decision tree, Maximum number of splits: 20, Number of learners: 30, Learning rate: 0.1, 5-Fold Cross Validation
Shallow Neural Network (SNN)	Number of fully connected layers = one hidden layer with size = 30, Activation: Sigmoid, Iteration limit: 1000, Standardize data: Yes, Regularization strength (Lambda): 0, 5-Fold Cross Validation
Bilayered Neural Network (BNN)	Number of fully connected layers: 2, First layer size: 10 Second layer size: 10, Activation: ReLU, Iteration limit: 1000, Regularization strength (Lambda): 0, Standardize data: Yes, 5-Fold Cross Validation
Logistic Regression Kernel (LRK)	Learner: Logistic Regression, Number of expansion dimensions: Auto, Regularization strength (Lambda): Auto, Kernel scale: Auto, Multiclass method: One-vs-One, Iteration limit: 1000, 5-Fold Cross Validation

**Table 6:** Summary of system evaluation results comparing performance of Kitsune and NSL-KDD datasets.

ML Models	CA%		MCR%		TC (#)		CS (Obs/Sec)	
	Kitsune	NSL-KDD	Kitsune	NSL-KDD	Kitsune	NSL-KDD	Kitsune	NSL-KDD
EBT	99.8	99.1	0.2	0.9	249	1332	90,000	84,000
ESK	99.4	98.4	0.6	1.6	780	2346	41	84
ERT	98.1	97.2	1.9	2.8	2495	4211	90,000	90,000
BNN	97.5	96.1	2.5	3.9	3250	5702	290,000	420,000
SNN	96.7	94.6	3.3	5.4	4290	8014	240,000	390,000
LRK	94.5	93.7	5.5	6.3	7215	9198	440	1900

**Table 7:** Summary of System Evaluation Results for EBT.

CA%	PR%	RC%	MCR%	FDR%	FNR%	TC (#)	CS (Obs/Sec)
99.8	99.7	98.1	0.2	0.9	1.7	249	90,000

**Table 8:** Comparison with other existing ML-based IoT-IDS systems.

Research	Year	ML Model	No. Classes	ACC%	PPV%	TPR%
Sarhan et al. [35]	2021	XRT Classifier	2–10	98.05	84.61	-
Ashraf et al. [36]	2021	STL Classifier	3	99.20	-	-
Kumar et al. [37]	2021	XGB Classifier	10	97.81	87.55	85.43
Khan et al. [38]	2021	HYB Classifier	7	76.00	75.00	75.00
Al-Haija et al. [39]	2020	S-CNN Classifier	5	98.20	98.27	98.20
Jinxin et al. [40]	2020	XGB Classifier	5	97.0	-	-
Alsaedi et al. [41]	2020	CART Classifier	9	77.00	77.00	77.00
Al-Haija et al. [60]	2020	S-CNN Classifier	2	99.30	99.33	99.18
Kumar et al. [42]	2019	kNN Classifier	3	94.44	92.00	100.0
Proposed model	2021	EDT Classifier	10	99.80	99.69	98.10
